# Manganite Heterojunction Photodetector with Broad Spectral Response Range from 200 nm to 2 μm

**DOI:** 10.3390/mi11020129

**Published:** 2020-01-23

**Authors:** Ru Chen, Zhiqing Lu, Kun Zhao

**Affiliations:** 1 College of New Energy and Materials, China University of Petroleum (Beijing), Beijing 102249, China; chenlingxs@163.com; 2 College of Sciences, China University of Petroleum (Beijing), Beijing 102249, China; hawklzqq@126.com

**Keywords:** manganite, heterostructure, photodetector

## Abstract

In this paper, we investigate the broad spectral photocurrent properties of the La_0.67_Ca_0.33_MnO_3_/Si (LCMO/Si) heterojunction from 200 nm to 2.0 μm, as the temperature increases from 95 to 300 K. We observed the junction’s uniform responsivity in the visible range and five absorption peaks at 940 nm, 1180 nm, 1380 nm, 1580 nm, and 1900 nm wavelengths. The temperature showed effective affection to the photocurrents at absorption peaks and the transition point occurred at 216 K, which was also displayed in the temperature dependence of junction resistance. On the basis of the results, we propose a possible model involving the quantum size effect at the junction interface as the mechanism. This understanding of the infrared photodetection properties of oxide heterostructures should open a route for devising future microelectronic devices.

## 1. Introduction

Oxide semiconductor devices based on the perovskite oxide films, whose properties can be controlled by magnetic field, electric field, and light irradiation, have attracted a great deal of interest. Experiments confirmed that manganite-based perovskite-type oxides have excellent ultraviolet (UV) photoresponse characteristics with ultrafast response-time of picosecond and high sensitivity, which makes this class of materials potentially useful for UV sensor applications [1,2]. Furthermore, manganite heterojunctions offer the features of tunability by magnetic and electric fields, high-sensitivity to light illumination and high carrier mobility, suggesting many possible applications and research directions including information storage, optoelectronics information processing, and advanced sample preparation techniques associated with microstructure modulate research [3,4,5,6,7]. In addition, similar to many optical materials with chemical stability, manganite heterojunctions are insensitive to harsh physical environment such as fluctuations of temperature and pressure, suggesting a potential application of manganite heterojunction photodetectors in harsh environments for the need of oil and gas optics [8,9,10,11,12]. Integrating the perovskite-type transition metal oxides with the silicon (Si)-based semiconductor technology would also introduce the possibility for a multifunctional microelectronic device [13,14,15].

Si photodetectors have already found wide acceptance for visible light applications, while it has small absorption coefficient in near-infrared (NIR) wavelength range because of the cut off wavelength of ~1100 nm. Now most NIR photodetectors were composed of PbS, PbSe, or InGaAs. The toxic precursors, such as Pb, Se, and As, was usually used to synthesize these materials. It is a meaningful thing to find non-toxic and pollution-free material for photodetector working at NIR wavelength range.

The infrared (IR) spectrum has become an important method to study the lattice distortion and been applied to investigate the photoconductive effect in perovskite manganese oxides, where mid-infrared or far-infrared spectra was used to explain the complex physical process in manganites such as the electronic transition, electron-phonon interaction, coupling between lattice, orbital, and spin, etc., [16,17,18,19,20,21,22]. In this paper the photocurrent response spectrum between 200 nm and 2 μm of the heterojunction La_0.67_Ca_0.33_MnO_3_/Si (LCMO/Si) is reported. The temperature dependence of the photocurrent response of the sample was investigated to reveal more information related to the photoelectric response, and selective absorption peaks were observed. The mechanism about the results is also discussed in the paper.

## 2. Materials and Methods

The LCMO/Si heterojunction was fabricated using the facing target sputtering technique. A 100 nm thickness LCMO layer was grown on a 0.5 mm thick n-type Si (001) wafer. The wafer temperature was kept at 680 °C with the oxygen pressure being 60 mTorr during deposition. Immediately after each deposition, the vacuum chamber was back-filled with 1 atm oxygen gas.

**Figure 1:**
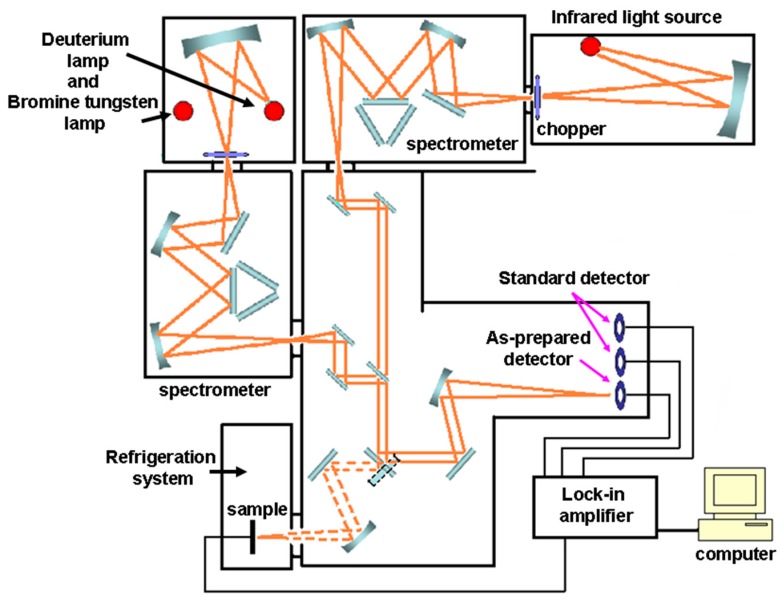
Spectral response measurement system.

The photocurrent of the sample was detected by the spectral response measurement system, as shown in Figure 1. The system was designed to measure the UV and IR spectral responsivity characteristics of samples in low temperature environment. The operation was automatically controlled, and the system maintained good closure during the measurement process. The selected all-reflected-light-route system, UV, visible light or IR, can be switched automatically with maximum light path coupling efficiency. The diameter of the light spot was 3 mm. The light intensity was calibrated using the spectrum of a commercial UV-100L Si photodiode (from OSI Systems Inc., Hawthorne, CA, USA) and the spectral responsivity was measured by a monochromator.

**Figure 2:**
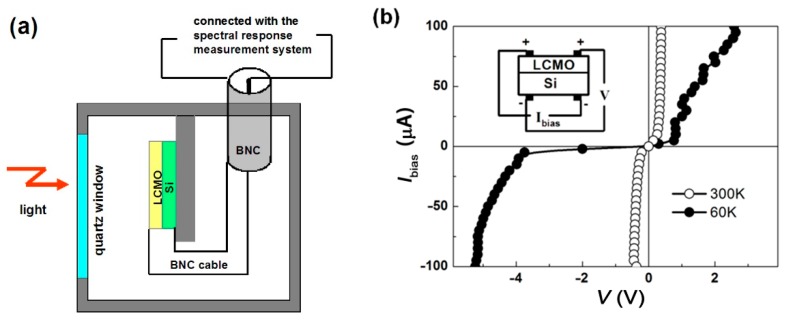
(**a**) The setup of La_0.67_Ca_0.33_MnO_3_/Si (LCMO/Si) heterojunction for the spectral response measurement. (**b**) The current-voltage curves of the LCMO/Si heterojunction at 300 and 60 K.

The LCMO/Si heterojunction for the photoelectric measurements was cut into 5 × 5 mm and two colloidal silver electrodes were prepared on the LCMO film and Si wafer. The sample was placed in an airtight holder with a quartz window and connected with the spectral response measurement system (Figure 2a). The typical current-voltage curves of the LCMO/Si heterojunction, shown in Figure 2b, were measured in the dark by tuning the applied voltage with a pulse-modulated voltage source at 300 and 60 K. The forward bias was defined as the current flowing from the upper LCMO layer to Si substrate. Thus the diodelike rectification characteristic can be ascribed to the presence of LCMO/Si interfacial potential because of the carrier diffusion.

## 3. Results and Discussions

**Figure 3:**
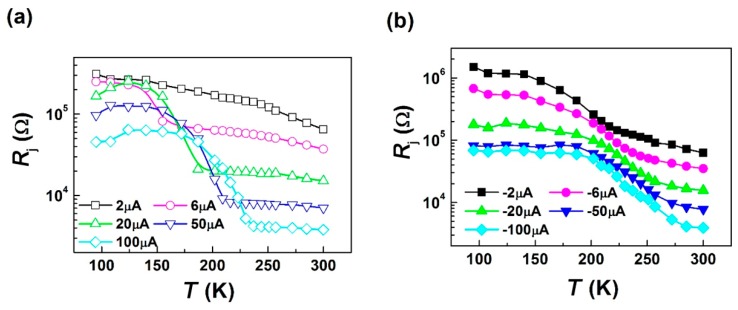
The temperature dependence of junction resistance *R*_j_ of a LCMO/Si junction under (**a**) the positive current bias and (**b**) the negative current bias.

The junction resistance *R*_j_ in LCMO/Si junction was measured with the temperature. As shown in Figure 3, *R*_j_ strongly depends on the bias, e.g., when the bias was turned from 20 μA to −20 μA *R*_j_ changed from 166.0 kΩ, 20.0 kΩ, and 15.1 kΩ to 178.1 kΩ, 98.2 kΩ, and 15.6 kΩ at 95 K, 202 K, and 300 K. In addition, taking the bias of 50 μA as an example, *R*_j_ decreased slightly from the beginning 95 K to 172 K and had a sharp change from 78 kΩ at 172 K to 8.2 kΩ at 216 K with a corresponding rate of 1.6 kΩ/K. Subsequently *R*_j_ maintained small change of about 0.01 kΩ/K till 300 K.

**Figure 4:**
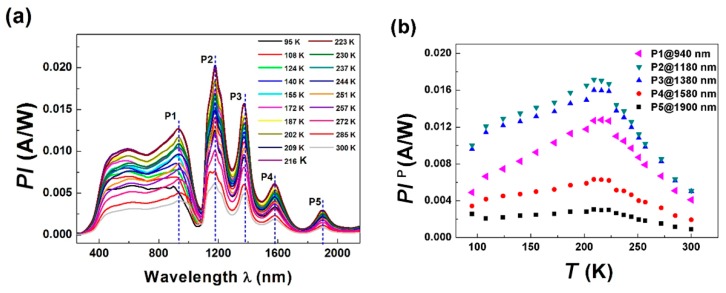
Photocurrent (*PI*) spectrum for various temperatures (**a**) and *PI* peaks (**b**) for 940 nm (P1), 1180 nm (P2), 1380 nm (P3), 1580 nm (P4), and 1900 nm (P5) of a LCMO/Si junction.

Figure 4a displays the photocurrent (*PI*) spectrum of the LCMO/Si junction under zero bias in the wavelength range of 200 nm < *λ* < 2200 nm. The junction’s responsivity was spectrally uniform in the visible range, while five absorption peaks P1, P2, P3, P4, and P5 were observed at *λ*_1_ = 1940 nm, *λ*_2_ = 1180 nm, *λ*_3_ = 1380 nm, *λ*_4_ = 1580 nm, and *λ*_5_ = 1900 nm wavelengths in each temperature because of the absorption characteristics of the LCMO/Si junction, and the peak value decreased with the increase of the wavelength. The temperature dependences of the photocurrent response *PI*^P^ at the five absorption wavelengths are shown in Figure 4b. *PI*^P^ monotonically increased from 0.0065 A/W, 0.012 A/W, 0.0115 A/W, 0.004 A/W, and 0.002 A/W at 108 K to 0.0122 A/W, 0.017 A/W, 0.0155 A/W, 0.006 A/W, and 0.0025 A/W at a turning point of 216 K and then dropped 0.004 A/W, 0.005 A/W, 0.005 A/W, 0.002 A/W, and 0.001 A/W at 300 K for selected wavelengths of *λ*_1_, *λ*_2_, *λ*_3_, *λ*_4_, and *λ*_5_.

Si cannot produce strong absorption features. Most of heterojunctions of manganite-based perovskite-type oxides exhibit the properties of p-n junction and quantum size effect can be produced when the thickness of the potential well material is about 50 nm thick. If quantum size effect occurs, the energy is quantized in the direction of the vertical interface, which will lead to the quantization of energy absorption in the material. Since a thin SiO_2_ layer of 3.6 nm thick exists in the LCMO/Si heterojunction [23], a quantum size effect was expected to occur. The interval of adjacent energy levels in an infinite quantum well is described as:

∆*E_n_*_,*n*+1_ = *π*^2^*η*^2^*m_n_*^−1^*d_w_*^−2^(*n* + 1/2) ∝ (*n* + 1/2) (1)

where *m_n_* is the electron effective mass and *d_w_* is the quantum well width. Thus, (∆*E_n_*_,*n*+1_ − ∆*E_n_*_+1,*n*+2_) is independent on *n* and

(∆*E_n_*_,*n*+1_−Δ*E_n_*_+1,*n*+2_) − (∆*E_n_*_+1,*n*+2_ − Δ*E_n_*_+2,n+3_) ≈ ∆*E_n_*_,*n*+1_ − 2∆*E_n_*_+1,*n*+2_ + ∆*E_n_*_+2,*n*+3_ (2)

As for present five special wavelengths *λ*_n_ (n = 1, 2, 3, 4, and 5),

(*λ*_1_^−1^ − *λ*_2_^−1^) − 2(*λ*_2_^−1^ − *λ*_3_^−1^) + (*λ*_3_^−1^ − *λ*_4_^−1^) ≈ 0.0034 (3)

and

(*λ*_2_^−1^ − *λ*_3_^−1^) − 2(*λ*_3_^−1^ − *λ*_4_^−1^)+(*λ*_4_^−1^ − *λ*_5_^−1^) ≈ 0.0030 (4)

Here, the above two similar data suggested that the present model involving the quantum size effect was adopted as the mechanism of IR photocurrent in LCMO/Si.

Noise performance is a critical factor for evaluating a detector. The noise current *I*_n_ is about 10^−4^ A/W in dark and is very low compared to the responsivity PI of the LCMO/Si junction when the light was on. The detectivity D* is determined by the ratio of *PI* and *I*_n_, and D* = *PI* (*fS*)^1/2^/*I*_n_, where *f* is the amplifier frequency bandwidth (500 MHz) and *S* is the detector area (~7.065 mm^2^). Thus D* is estimated to be about 2.38 × 10^3^ Hz^1/2^m, 2.97 × 10^3^ Hz^1/2^m, 2.97 × 10^3^ Hz^1/2^m, 1.19 × 10^3^ Hz^1/2^m, and 0.59 × 10^3^ Hz^1/2^m at 300 K for selected wavelengths of *λ*_1_, *λ*_2_, *λ*_3_, *λ*_4_, and *λ*_5_, suggesting that the LCMO/Si junction could be well-suited as an IR detector.

Perovskite-type oxides detectors possess a number of significant characteristics, and are ideally suited to detect small changes in a relatively large background level of incident energy, which can be used over a large spectral bandwidth. Here it has been shown that a specific manganite heterojunction has the ability to be an IR detector since it can produce photocurrent in the IR regime. The devices have a number of important characteristics (low cost, low power, good performance, wide operating range of temperature, a high degree of environmental stability, and reliability) which make them ideal for a range of applications from consumer and commercial to military requirements. LCMO/Si junction is a new material for photodetector fabrication compared to traditional materials. It is anticipated that manganite heterojunction IR detectors will assume an ever growing importance in our society over the next few years.

## 4. Conclusions

In conclusion, we fabricated a manganite-based heterojunction by depositing a LCMO thin film on the Si substrate. The broad spectral photocurrent effect of the junction was systematically studied in a temperature range from 95 to 300 K. The responsivity of LCMO/Si heterojunction was spectrally uniform in the visible range. Five absorption peaks occurred at 940 nm, 1180 nm, 1380 nm, 1580 nm, and 1900 nm in the IR range, which is explained in terms of a quantum size effect model since an interface existed in the present photodetector. However, relative contributions from individual interface are still not clear and further studies is needed to clarify the *PI* mechanisms.
